# Intermediarios protésicos en implantología oral. Revisión de la literatura

**DOI:** 10.21142/2523-2754-0902-2021-064

**Published:** 2021-06-21

**Authors:** César-Augusto Padilla-Avalos, Consuelo Marroquín-Soto

**Affiliations:** 1 Facultad de Odontología, Universidad de San Martín de Porres. Lima, Perú. cesarpadilla160@gmail.com, marroquinconsuelo@gmail.com Universidad de San Martín de Porres Facultad de Odontología Universidad de San Martín de Porres Lima Peru cesarpadilla160@gmail.com marroquinconsuelo@gmail.com

**Keywords:** diseño de implante dental-pilar, prótesis dental de soporte implantado, Dental Implant-Abutment Design, Implant-Supported Dental Prosthesis

## Abstract

Los intermediarios protésicos en implantología oral son los aditamentos que permiten la conexión entre el implante y la prótesis propiamente dicha. Aunque dicha rehabilitación posee una alta tasa de éxito, la selección del pilar protésico representa una fase importante en el tratamiento implantológico. Actualmente, existe una gran variedad de pilares intermediarios, que corresponden a las diversas técnicas y materiales. Los intermediarios protésicos se podrían clasificar según el tipo de conexión, su retención a la prótesis, su relación axial con el cuerpo del implante, su material de fabricación, su tipo de fabricación o si son para rehabilitación unitaria o múltiple. Esta situación puede generarle dudas al rehabilitador al momento de elegir los aditamentos implantológicos idóneos para cada caso en particular, lo que genera un escenario de dilema al seleccionar el aditamento protésico que permita lograr una rehabilitación satisfactoria: funcional, estética y que preserve los principios biológicos. En efecto, la implantología oral ha revolucionado la odontología y seguirá ampliando el abanico de posibilidades; por ello, es importante clasificar las opciones protésicas disponibles. La presente revisión de la literatura tiene como objetivo evidenciar las diferentes alternativas y opciones de pilares intermediarios más utilizados en prótesis sobre implantes.

## Abstract (en)

Prosthetic intermediaries in oral implantology are attachments that allow connection between the implant and the prosthesis itself. Although this rehabilitation has a high success rate; the selection of the prosthetic abutment represents is important in implant treatment. Currently, there is a great variety of intermediate abutments, corresponding to various techniques and materials. Prosthetic intermediaries can be classified according to the type of connection, their retention to the prosthesis, their axial relationship with the implant body, the manufacturing material and type of manufacture, or whether rehabilitation is single or multiple. This scenario can lead to doubts as to the selection of the ideal implant attachment in each case in order to achieve satisfactory rehabilitation, as well as functional and aesthetic requirements and the preservation of biological principles. Indeed, oral implantology has revolutionized dentistry and will continue to expand the range of possibilities; therefore, it is important to classify the prosthetic options available. The present literature review aims to demonstrate the different alternatives and options available for the intermediate abutments most used in implant prostheses.

## INTRODUCCIÓN

La rehabilitación sobre implantes en pacientes total o parcialmente edéntulos posee una alta tasa de éxito. Son considerados tratamientos predecibles, viables y duraderos gracias al avance de la implantología en los últimos años ^1^. A su vez, brindan beneficios como mejorar la calidad de vida y aumentar la satisfacción del paciente ^2^. 

En la actualidad, existe gran número de empresas que fabrican implantes, cada uno con diferentes componentes o variaciones que los hacen únicos. Este escenario coloca al rehabilitador en el dilema de seleccionar qué aditamentos protésicos son los adecuados para lograr la rehabilitación sobre implantes de manera satisfactoria ^3^.

Los intermediarios o pilares protésicos son aditamentos empleados para la rehabilitación sobre implantes, y corresponden a estructuras de conexión entre el implante y la restauración que son retenidos al implante por un tornillo o mediante fricción ^4^^,^^5^. La selección del pilar protésico para cada caso individual de paciente es una fase importante del tratamiento implantológico. Los estudios clínicos que evalúan las prótesis fijas implantosoportadas a largo plazo evidencian bajas tasas de complicaciones técnicas con respecto al pilar intermediario en sí ^6^.

La literatura reporta diversas clasificaciones de los intermediarios: según el tipo de conexión de la plataforma del implante, según el tipo de indicación para prótesis cementada o atornillada, según su modo de fabricación, según la retención de la prótesis, según el material de fabricación, entre otras ^3^^,^^5^^,^^7^^,^^8^ (Tabla 1).

**Tabla 1:** Clasificación de intermediarios protésicos en implantología oral

Intermediarios según clasificación	
Tipo de conexión	Intermediarios de conexión externa Intermediarios de conexión interna
Retención de la prótesis	Intermediarios para prótesis atornillada Intermediarios para prótesis cementada
Relación axial con el cuerpo del implante	Intermediarios rectos Intermediarios angulados
Material de fabricación	Titanio Oro Acero quirúrgico inoxidable Poliéter éter cetona Zirconio UCLA
Tipo de fabricación	Intermediarios prefabricados Intermediarios modificables
Utilizados en prótesis múltiple	Intermediarios de multiunit Intermediarios de pilar en bola

El objetivo de la presente revisión de la literatura es evidenciar las alternativas y opciones de pilares intermediarios utilizados en prótesis sobre implantes, a fin de describir sus características, diferencias e indicaciones.

## METODOLOGÍA

Para el desarrollo de este artículo de revisión de literatura, se realizó la búsqueda bibliográfica en Medline vía PubMed y LILACS, empleando las siguientes palabras claves y frases: “pilares de implante”, “intermediarios protésicos” y “aditamentos protésicos”. Se seleccionó la literatura más relevante y vigente hasta el 31 de diciembre de 2020, y el análisis se enfocó en evidenciar la clasificación de los intermediarios según el tipo de conexión, retención de la prótesis, relación axial al cuerpo del implante, material y tipo de fabricación. Se encontraron 45 artículos potencialmente relevantes, pero solo se incluyeron en el estudio 31 debido al impacto académico que presentaron.

## CLASIFICACIÓN DE INTERMEDIARIOS

### INTERMEDIARIOS SEGÚN EL TIPO DE CONEXIÓN

La elección del tipo de conexión (de la restauración al implante a través del pilar atornillado en la plataforma) es una de las decisiones más importantes en la rehabilitación sobre implantes ^9^. Los diseños de conexión entre el implante y el pilar intermedio pueden clasificarse en dos grupos principales: externa e interna. 

#### Intermediarios en conexión externa

La conexión externa fue el primer sistema adoptado en implantología por Branemark y se basa en el diseño de un hexágono de 0,7 mm de altura; sin embargo, ha sido modificada en diámetro y altura. Su diseño ofrece una gran variedad de opciones de restauración debido a la versatilidad de pilares intermediarios entre los fabricantes. Esta conexión es adecuada en el procedimiento quirúrgico de dos pasos, ya que facilita la segunda etapa y la fase con el pilar de cicatrización. En segundo lugar, simplifica el registro de la conexión externa en la impresión y la fase protésica debido a su ajuste y compatibilidad con diferentes soluciones protésicas ^10^^,^^11^.

En la actualidad, aunque este tipo de conexión es utilizado ampliamente, tiene algunos inconvenientes debido a su limitación en altura, que lo hace ineficaz cuando se aplica una carga axial excesiva. A su vez, está asociado con desventajas como el micromovimiento y aflojamiento del tornillo del pilar intermedio entre el 6% y el 48% de los casos según estudios ^12^, por lo que se vincula con complicaciones mecánicas y biológicas, y está contra indicado en ciertas situaciones clínicas ^3^^,^^13^^,^^14^.

#### Intermediarios en conexión interna

El objetivo de este diseño es mejorar y aumentar la estabilidad de la conexión a lo largo de la función cuando la prótesis es sometida a cargas. Estudios clínicos a largo plazo indican que la conexión interna fue diseñada para reducir las complicaciones encontradas en el otro tipo de conexión ^3^^,^^13^.

Dentro de la conexión interna, surge una variante llamada cono Morse, término creado en la industria de las herramientas mecánicas que corresponde a un mecanismo de encastre por fricción, lo que permite un encaje impermeable, caracterizado por su fijación antirrotacional estable y su alta resistencia mecánica a las fuerzas ^15^. Este diseño está dirigido a reducir la incidencia de pérdida ósea por la penetración de bacterias a la interfase implante-intermediario, debido a una brecha marginal menor frente a otros sistemas ^16^.

Sailer desarrolló una revisión sistemática de estudios clínicos y de laboratorio, y evidenció una tendencia de mejor funcionamiento y menor aflojamiento del tornillo con los pilares intermediarios de conexión interna ^17^.

### INTERMEDIARIOS SEGÚN SU RETENCIÓN A LA PRÓTESIS

El método ideal para la retención de la prótesis al pilar intermediario es aquel que permita pasividad en la colocación, optimice la dirección de las cargas, mejore la estética, permita el fácil acceso al procedimiento de prueba y reduzca tanto la pérdida de cresta ósea como las complicaciones, el costo y el tiempo operatorio ^18^. 

La selección del sistema de retención (prótesis atornillada o cementada) requiere la evaluación de algunos criterios, y posiblemente el más relevante es el tipo de conexión protésica. En conexiones hexagonales, como en el hexágono externo (HE) o el interno (HI), las tensiones promovidas por la carga oclusal se transfieren directamente a los tornillos de fijación, mientras que en las conexiones internas de cono Morse (CM) se distribuyen estas tensiones por todo el implante, lo cual favorece una mayor estabilidad y un menor riesgo de problemas mecánicos como aflojamiento de tornillos o fracturas en la unión entre implante y pilar intermedio ^5^^,^^8^.

Es por ello que la elección entre una prótesis atornillada y cementada ha sido un tópico muy debatido en implantología. El conocimiento del mecanismo entre ambos tipos, ayudará al rehabilitador a seleccionar la prótesis ideal para cada caso clínico, a fin de lograr resultados estéticos finales ^3^. 

Los factores relacionados con los diferentes métodos de retención de las prótesis sobre implantes son los siguientes: facilidad de fabricación y costo, estética, acceso, oclusión, retención, incidencia de pérdida de retención, recuperabilidad, pasividad de ajuste, restricción de la posición del implante, efecto sobre la salud del tejido periimplantario, provisionalización, carga inmediata, procedimientos de impresión, fractura de porcelana y rendimiento clínico ^1^.

#### Intermediarios en prótesis atornillada

La prótesis atornillada requiere de poco espacio interoclusal (4 mm) sin perder su retención debido a los aditamentos protésicos que la conforman. No requiere el uso de cemento para su fijación y posee alta tasa de reversibilidad, lo que permite la sustitución periódica de componentes protésicos cuando sea necesario, reajuste oclusal y acceso en situaciones de fractura o complicaciones en los tornillos de fijación. Además, esta característica permite una mejor higiene y preservación de los tejidos periimplantarios ^7^^,^^8^.

El sistema de intermediarios para prótesis atornillada, en la mayoría de las marcas comerciales, es denominado *multiunit*. Estos pilares intermediarios son indicados en prótesis atornilladas unitarias y múltiples, ya que proporcionan un ajuste absolutamente pasivo de la prótesis, incluso con una divergencia significativa de los ejes de los implantes colocados. Debido a que todas las manipulaciones se realizarán por encima del nivel del hueso y la plataforma del implante, la colocación de estos pilares protege los tejidos blandos periimplantarios de múltiples daños causadas por supraestructuras de implantes atornilladas/desenroscadas ^19^.

#### Intermediarios en prótesis cementada

La fabricación de prótesis cementada sobre implantes es menos compleja que las atornilladas, debido a que utilizan técnicas prostodónticas clínicas y de laboratorio convencionales, propias de las restauraciones cementadas ^1^. Este tipo de prótesis requiere de un componente vertical de al menos 5 mm para no perder retención, altura que fue documentada para alcanzar una retención predecible. Existen diversos factores que afectan la retención de la prótesis cementada, como la conicidad, el área o altura de la superficie, y la rugosidad del pilar intermedio ^18^^,^^20^. La prótesis cementada tiene superficies oclusales intactas y esta característica implica una mejor dirección de las fuerzas oclusales a lo largo del eje del implante, ya que los contactos oclusales ocurren directamente sobre la corona y no sobre el material restaurador obliterante del orificio de acceso a los tornillos, como ocurre en las restauraciones atornilladas ^21^.

En situaciones clínicas, cuando existe un posicionamiento inadecuado de implantes en dirección vestibulolingual, mesiodistal e incluso coronoapical, el profesional debe evaluar aspectos como la presencia adecuada de tejido gingival, la línea de sonrisa del paciente y el espacio interoclusal existente para elegir la mejor opción de pilar intermediario y el consiguiente sistema de retención protésica para cada caso en particular ^8^.

**Figura 1:**
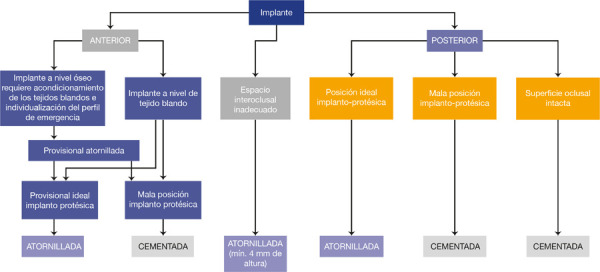
Árbol de decisiones para la indicación de prótesis fija atornillada versus cementada implantosoportada (adaptado de Wittenebe *et al*.^22^

Las recomendaciones clínicas para prótesis atornilladas son contar con espacio interoclusal mínimo (4 mm), en prótesis con diseño en voladizo o de largo alcance, para evitar el posible remanente de cemento; en zona estética, para provisionalización de implantes que permitan el acondicionamiento de tejidos blandos y el perfil de emergencia, y cuando se desea reversibilidad. Sin embargo, la prótesis cementada se indica en tramos cortos con márgenes al nivel o encima de la mucosa, para compensar implantes mal inclinados cuando se desea un mejor control de la oclusión sin un orificio de acceso ^22^ (Figura 1).

### INTERMEDIARIOS SEGÚN SU RELAXIÓN AXIAL CON EL CUERPO DEL IMPLANTE

Según el eje axial del cuerpo del implante, los pilares intermediarios pueden ser rectos o angulados.

#### Intermediarios rectos

Son pilares intermediarios que no presentan angulación con respecto al eje axial del implante, solo varía el *cuff* y se presentan para conexión interna o externa. Este pilar, conformado por un hexágono tipo hembra, se conecta al implante mediante un parafuso de fijación ^7^.

#### Intermediarios angulados

Estos pilares intermediarios comparten las mismas características que los pilares rectos; sin embargo, su angulación podría ser de 17º o 30º con respecto al eje axial del implante. Se suele indicar para resolver problemas de angulación y mejorar la posición final del tornillo de acceso ^7^.

### INTERMEDIARIOS SEGÚN SU MATERIAL DE FABRICACIÓN

Existe una amplia variedad de materiales con los que se confeccionan los pilares intermediarios, lo cual se convierte en un desafío para el rehabilitador que busca comprender la respuesta biológica a cada material, así como la mejor indicación para cada uno de los tipos. El sello de mucosa que rodea al pilar intermediario de un implante dental es un factor esencial e importante para prevenir la colonización por parte de bacterias del hueso crestal y alrededor del cuello del implante. Por lo tanto, es importante conocer la anatomía del sello de mucosa para comprender la respuesta de los tejidos blandos ^23^.

#### Intermediarios de titanio

El titanio, como aleación, se presenta en grado I (comercialmente puro) o grado V. Se caracteriza por su excelente estabilidad a largo plazo y su alta supervivencia en restauraciones soportadas por pilares de titanio, según diversos trabajos clínicos ^7^. Diversos estudios de seguimiento sustentan los resultados y el mantenimiento favorable en tejidos blandos con pilares de titanio (mecanizado o pulido), por lo que se trata del material para intermediario más validado en la literatura ^22^.

Algunos pilares intermediarios fabricados con titanio Laser-Lok presentan una mayor capacidad para unirse con el tejido conectivo en comparación con todos los demás materiales. Este sello periimplantario permite un mejor mantenimiento de los tejidos blandos a largo plazo ^22^.

#### Intermediarios de oro

El oro es una aleación noble, con excelente biocompatibilidad, muy bajo nivel de corrosión, buenas propiedades mecánicas y reducción del coeficiente de fricción entre los aditamentos protésicos ^7^. Sin embargo, la literatura reporta discrepancias sobre la capacidad de formar un sello periimplantario adecuado y su mantenimiento a largo plazo. 

Los intermediarios de oro se utilizaron en la confección de prótesis en busca de la estética, en casos de altura reducida para el espacio oclusal vertical o ángulos personalizados. Los pilares intermediarios de oro fundido fueron muy utilizados durante las décadas de 1980 y 1990, pero, con la introducción de pilares originales más sofisticados y pilares fresados CAD/CAM, han perdido popularidad ^23^. 

#### Intermediarios de acero inoxidable quirúrgico

El acero quirúrgico inoxidable es un material utilizado en instrumental médico e incluye elementos de aleación de cromo, níquel y molibdeno. El cromo proporciona resistencia al rayado y a la corrosión, mientras que el níquel proporciona un acabado pulido y liso. Por otro lado, el molibdeno le da mayor dureza, lo que ayuda a mantener el filo. El acero inoxidable es de fácil limpieza y esterilización, y es resistente a la corrosión. Las aleaciones de níquel/cromo/molibdeno, a veces, se usan para pilares intermediarios de implantes, pero la reacción del sistema inmunológico al níquel es una complicación potencial. El acero inoxidable de grado quirúrgico se puede utilizar únicamente para pilares de implantes temporales, mas no como un material definitivo ^23^.

#### Intermediarios de poliéter éter cetona (PEEK)

Este material se utiliza con habitual frecuencia para la fabricación de pilares intermediarios provisionales, debido a que su permanencia en boca no puede exceder los 180 días. El PEEK es un polímero orgánico de color beige o blanco, y un termoplástico semicristalino con excelentes propiedades de resistencia mecánica y química. Es altamente resistente a la degradación térmica y a la humedad. Estas robustas propiedades han convertido al PEEK en un material ideal para pilares provisionales ^23^.

#### Intermediarios de zirconio

El dióxido de zirconio (ZrO2), conocido como zirconio, es un óxido cristalino blanco de este mineral. Los avances en la ciencia de los biomateriales y la tecnología de fabricación de cerámica han permitido la producción de zirconio biocompatible y de alta resistencia que se puede utilizar en dispositivos biomédicos y pilares intermediarios de implantes. Debido a sus propiedades y resistencia, se utiliza siempre que las consideraciones estéticas sean exigentes ^23^^-^^25^. 

Los pilares intermediarios de zirconio proporcionan una mejor adaptación e integración del color y la superficie de los tejidos blandos adyacentes, y están especialmente indicados en pacientes con mucosa periimplantaria fina. Sin embargo, las fracturas de los pilares intermediarios de zirconio todavía son frecuentes; por lo tanto, las indicaciones deben limitarse hasta 20 o 30 grados de angulación para prevenirlas ^25^.

#### Intermediarios UCLA

Este pilar permite la confección de la restauración directamente en la fijación del implante, sin pasar por el cilindro del pilar transmucoso. Inicialmente, este intermediario resolvió los problemas causados por la falta de espacio interoclusal cuando se cuenta con reducido espacio para la restauración, ya que elimina el uso de más aditamentos protésicos. Este pilar puede ser utilizado tanto en pacientes parcialmente edéntulos como en barras para sobredentaduras ^26^.

### INTEMEDIARIOS SEGÚN SU TIPO DE FABRICACIÓN

Un pilar intermediario protésico exitoso debe soportar la mucosa periimplantaria; por lo tanto, la restauración debe estar provista de contornos óptimos y un perfil de emergencia adecuado. Consecuentemente, esto brindará un resultado final de una prótesis funcional, estética y fácil de higienización. El proceso de diseño, fabricación de contornos y acabado de los pilares intermediarios son factores que influyen directamente en los resultados estéticos ^27^.

Los intermediarios según su tipo de fabricación, pueden ser pilares prefabricados o mecanizados.

#### Intermediarios prefabricados

No modificables: Son los aditamentos fabricados con el mismo torno alfanumérico que los implantes dentales; estos poseen alto ajuste, adaptación marginal y son comercializados por las mismas compañías. Presentan una amplia gama de opciones protésicas, con el fin de resolver las diferentes situaciones clínicas durante la rehabilitación sobre implantes ^7^.

Modificables: Estos intermediarios de titanio son fresables y pueden ser modificados en su diámetro y forma, según la necesidad del caso, para conformar un adecuado perfil de emergencia ^7^^,^^27^.

#### Intermediarios fabricados en laboratorio

Los pilares intermediarios también se pueden producir mediante la tecnología de diseño y fabricación asistido por computadora (CAD/CAM). El proceso CAD/CAM controla de forma óptima la geometría del intermediario, incluyendo la posición de la prótesis tomando en cuenta las raíces naturales vecinas y el margen gingival, reduciendo posteriormente el riesgo de dejar restos de cemento en el surco. El acabado del pilar está controlado, lo que evita bordes afilados y el diseño puede compensar la mala angulación del implante ^24^.

### INTERMEDIARIOS EN PRÓTESIS MÚLTIPLE

#### Intermediarios multiunit

Este sistema de intermediarios permite múltiples prótesis implatosoportadas atornilladas, ya que proporcionan un eje pasivo de la restauración, inclusive con una divergencia significativa de los los implantes colocados. Adicionalmente, protegen los tejidos blandos periimplantarios de daños causados por múltiples supraestructuras atornilladas a los implantes, ya que toda manipulación se realiza por encima de la plataforma del implante y, por ende, del nivel del hueso ^19^.

#### Intermediarios de pilar de bola

Consiste en un sistema de mecanismo de fijación extracoronal, el cual es utilizado para retener una sobredentadura. Consiste en un intermediario de forma esférica, que encaja en una carcasa metálica de fijación. El uso de este pilar permite un reemplazo fácil de componentes y se asocia con una menor tensión sobre el implante. Cabe mencionar que son fabricados en una variedad de materiales y el que se seleccione puede depender de la naturaleza del procedimiento y del tipo de implante ^28^.

## DISCUSIÓN

La presente revisión de la literatura resume la clasificación actual de los tipos de intermediarios protésicos en rehabilitación sobre implantes, lo cual otorga un amplio panorama a los profesionales sobre las opciones vigentes para la toma de decisiones clínicas basadas en el diagnóstico y planificación de cada caso en particular. 

Respecto de la selección del tipo de conexión del implante-pilar suele basarse en la experiencia clínica del profesional. Sin embargo, el sistema de conexión implante-pilar puede considerarse un factor que influye en la remodelación ósea alrededor de los implantes después de la carga funcional ^29^. Araujo *et al.*^13^ realizaron una revisión sistemática donde analizaron si la conexión implante-pilar influye en la magnitud de la pérdida ósea, con lo que hallaron que los implantes de conexión interna se asociaron con una menor pérdida ósea que los implantes de conexión externa. 

Es pertinente mencionar algunas situaciones clínicas para facilitar la elección de estos aditamentos protésicos. En casos de rehabilitaciones de arco completo, es preferible que la prótesis sea retenida con tornillos y se indicará intermediarios atornillados, porque las complicaciones en estas prótesis extensas son más comunes que en las de menor extensión ^30^. De igual manera, en pacientes que tienen un alto riesgo de desarrollar recesión gingival, es recomendable planificar la rehabilitación con prótesis atornilladas y, consecuentemente, se optará por intermediarios atornillados, con el fin de permitir su remoción sin complicaciones y la posterior modificación de la prótesis de acuerdo con la nueva situación. No obstante, en situaciones donde la superficie oclusal de la restauración se ve comprometida con respecto a la estética o la estabilidad oclusal (debido a la presencia de un material que selle el acceso al tornillo), se optará por prótesis cementadas y los intermediarios específicos para esos casos ^1^^,^^31^.

Sobre la técnica de fabricación, en una revisión realizada por Long *et al.*^27^, se encontró que solo los intermediarios elaborados por CAD/CAM presentan una alta tasa de supervivencia en seguimiento de 1 y 3 años. Para las investigaciones que compararon intermediarios CAD/CAM con convencionales, no hubo diferencias significativas en la mayoría de los resultados clínicos al año de seguimiento. Sin embargo, Schepke *et al.*^24^ realizaron un ensayo clínico en el que llegan a la conclusión que el uso de un pilar de zirconio personalizado por CAD/CAM para reemplazar un diente no se asocia con una mejora del resultado que reflejen el desempeño clínico, la alteración ósea periimplantaria, la satisfacción o el grado en que los pacientes se cumplen las expectativas en comparación con el uso de un pilar estándar.

## CONCLUSIONES

La revisión del tema tratado presenta la clasificación de los pilares intermediarios según sus indicaciones, características y limitaciones. La selección depende del tipo de conexión, su retención a la prótesis, su relación axial con el cuerpo del implante, su material de fabricación y su tipo de fabricación, sean restauraciones individuales o múltiples, provisionales o definitivas.

Se recomienda analizar cada situación clínica de manera particular. La planificación protésica de implantes inicia con el diagnóstico médico-odontológico integral de cada paciente (clínico, laboratorial e imagenológico). Para entonces, se deben realizar evaluaciones clínicas y tomográficas del complejo estomatognático y, basados en la expectativa del paciente, sugerir la selección del pilar intermediario idóneo (según cada sistema de implantes) y el tipo de prótesis por utilizar.

Actualmente, las compañías de implantes disponen de una amplia variedad de opciones protésicas (según diseños, tipos y materiales de pilares) para llevar a cabo casos clínicos de diferente nivel de complejidad, y es responsabilidad del clínico la planificación de caso y la evaluación exhaustiva para la selección de aditamentos y la obtención de resultados satisfactorios, que preserven los principios biomecánicos y biológicos. Sobre todo, se recomienda evitar utilizar “aditamentos genéricos” o probar la compatibilidad con otras marcas.

## Fuente de financiamiento:

Los autores financiaron la investigación
